# Multi-Strategy Improved Harris Hawk Optimization Algorithm and Its Application in Path Planning

**DOI:** 10.3390/biomimetics9090552

**Published:** 2024-09-12

**Authors:** Chaoli Tang, Wenyan Li, Tao Han, Lu Yu, Tao Cui

**Affiliations:** School of Electrical & Information Engineering, Anhui University of Science and Technology, Huainan 232001, China; chltang@mail.ustc.edu.cn (C.T.); than@aust.edu.cn (T.H.); 2021200747@aust.edu.cn (L.Y.); 2022200921@aust.edu.cn (T.C.)

**Keywords:** Harris Hawk Optimization algorithm, double adaptive weight strategy, Dimension Learning-Based Hunting search strategy, Dung Beetle Optimizer algorithm, path planning

## Abstract

Path planning is a key problem in the autonomous navigation of mobile robots and a research hotspot in the field of robotics. Harris Hawk Optimization (HHO) faces challenges such as low solution accuracy and a slow convergence speed, and it easy falls into local optimization in path planning applications. For this reason, this paper proposes a Multi-strategy Improved Harris Hawk Optimization (MIHHO) algorithm. First, the double adaptive weight strategy is used to enhance the search capability of the algorithm to significantly improve the convergence accuracy and speed of path planning; second, the Dimension Learning-based Hunting (DLH) search strategy is introduced to effectively balance exploration and exploitation while maintaining the diversity of the population; and then, Position update strategy based on Dung Beetle Optimizer algorithm is proposed to reduce the algorithm’s possibility of falling into local optimal solutions during path planning. The experimental results of the comparison of the test functions show that the MIHHO algorithm is ranked first in terms of performance, with significant improvements in optimization seeking ability, convergence speed, and stability. Finally, MIHHO is applied to robot path planning, and the test results show that in four environments with different complexities and scales, the average path lengths of MIHHO are improved by 1.99%, 14.45%, 4.52%, and 9.19% compared to HHO, respectively. These results indicate that MIHHO has significant performance advantages in path planning tasks and helps to improve the path planning efficiency and accuracy of mobile robots.

## 1. Introduction

With the gradual intelligentization of modern industry, mobile robots have attracted more and more attention in many fields and have been widely used in industry, medical treatment, agriculture, the service industry, and other fields. As a key technology for the autonomous navigation of mobile robots, path planning can be used to plan a collision-free path from the starting point to the endpoint for the robot in a complex environment [1]. However, existing path planning algorithms still face many challenges, especially in terms of solution accuracy, convergence speed, and avoiding falling into local optimization.

Path planning algorithms can be divided into traditional algorithms and swarm intelligence algorithms. Traditional algorithms include the Dijkstra algorithm [2], A-Star algorithm [3], and Artificial Potential Field (APF) [4]. The A-Star algorithm can find a fast and accurate shortest path solution through heuristic functions, but it has a large dependence on heuristic functions and a high memory consumption. The Dijkstra algorithm is simple to implement, but it is less efficient when dealing with maps containing a large number of nodes. The Artificial Potential Field method is simple and computationally efficient, making it suitable for real-time path planning, but it easily falls into local minima and has difficulty coping with complex environments.

The swarm intelligence algorithm forms a self-organizing adaptive stochastic optimization algorithm with bionic behavior by observing the living habits, foraging behavior, and social characteristics of the population [5]. Compared with traditional algorithms, group intelligence algorithms do not require gradient information, and they are flexible and easy to implement [6]. They are versatile and simple to implement and have high optimization efficiency and excellent performance in solving complex problems. By simulating the collaboration and competition mechanism of biological groups, the group intelligence algorithm shows great flexibility and adaptability, making them powerful complements to traditional algorithms. Due to these advantages, group intelligence algorithms show a wide range of application prospects and have potential applications in various fields.

Because of its better robustness, flexibility, and high search accuracy, the swarm intelligence algorithm has been widely used in path planning. Common swarm intelligence algorithms for path planning include the Whale Optimization Algorithm (WOA), Grey Wolf Optimizer algorithm (GWO), Ant Colony Optimization algorithm (ACO), Sparrow Search Algorithm (SSA), etc. Tian et al. [7] proposed a hybrid Firefly–Whale Optimization Algorithm (FWOA) combining multiple swarms and dyadic learning strategies by simulating whale foraging behaviors and firefly blinking characteristics, which can quickly find the optimal in complex mobile robot working environments and significantly improve accuracy and efficiency when solving the path planning problem. Yu et al. [8] proposed a hybrid Grey Wolf Optimizer algorithm and Differential Evolution algorithm to solve the UAV path planning problem; the improved algorithm can make the path shorter and smoother. Wu et al. [9] proposed a Modified Adaptive Ant Colony Optimization algorithm (MAACO), which can effectively reduce the path length and the number of turns and improve the convergence speed. He et al. [10] proposed an improved chaotic Sparrow Search Algorithm to overcome the problems of slow convergence and easily falling into local optimization in UAV path planning for 3D complex environments. However, these algorithms still suffer from the problems of insufficient solution accuracy and a slow convergence speed and can easily fall into local optimization in path planning, which limits their applications in more complex scenarios.

The Harris Hawks Optimization (HHO) algorithm is a swarm intelligence optimization algorithm proposed by Heidari et al. in 2019 [11]. This algorithm simulates the hunting behavior of Harris hawks to achieve the purpose of optimizing the search. Compared with other intelligent algorithms, it has the advantages of fewer parameters, simple operation, simple principles, and strong versatility. At present, the HHO algorithm has been widely used in scheduling problems [12], image processing [13], power system configuration [14,15], neural network training [16], and path planning [17,18]. However, the HHO algorithm also faces the problems of poor solution accuracy, a slow convergence speed, and the tendency to fall into local optimization. To solve these problems, researchers have proposed a variety of improvement strategies. Qu et al. [19] proposed an HHO algorithm with an information exchange function. By exploring the shared area, information exchange and sharing between Harris hawks are realized, and the convergence accuracy and efficiency are improved. Jiao et al. [20] combined orthogonal learning and reverse learning to effectively and accurately estimate the parameters of solar cells and photovoltaic modules. Zou et al. [21] improved the development phase of the HHO algorithm by using differential perturbation and a greedy strategy to enhance population diversity. In addition, a new conversion strategy was used to make the algorithm flexibly switch between search and development, thereby enhancing its global search ability. Li et al. [22] proposed an exploration strategy based on logarithmic spiral and reverse learning to improve the exploration ability of HHO. Hussain et al. [23] integrated the sine and cosine algorithm to enhance the invalid search of the HHO algorithm and avoided the stagnation of the solution of the HHO algorithm by dynamically adjusting the strategy.

HHO is also widely used in path planning. Li et al. [24] proposed a path planning algorithm based on a bionic neural network and improved HHO, which has a periodic energy decline adjustment mechanism and has solved the problem of multi-UAV path planning in a three-dimensional space. Belge et al. [17] used a hybrid HHO-GWO meta-heuristic optimization algorithm for path planning and tracking to avoid obstacles. Huang et al. [25] introduced an improved sine-trend search strategy and nonlinear jump intensity into the HHO algorithm. The improved algorithm has good feasibility and effectiveness in trajectory planning. Aiming to solve problem of multi-robot path planning, Nasr AA [26] proposed a fusion algorithm using K-means clustering and HHO, which effectively improved the convergence speed of the algorithm and reduced the calculation amount of the algorithm. Dehkordi AA et al. [27] introduced chaotic mapping into the HHO algorithm to ensure the uniform distribution of the initial population. In the path optimization problem of the Internet of Vehicles, the improved algorithm has been proven to have significantly improved convergence speed and accuracy.

To address these problems faced by the HHO algorithm in path planning, this paper proposes a Multi-strategy Improved Harris Hawk Optimization (MIHHO), which aims to improve the algorithm’s solution accuracy, convergence speed, and ability to avoid local optima. The main contributions of this paper include the following:

1. Double adaptive weight strategy: In MIHHO, the convergence accuracy and speed of path planning are effectively improved by enhancing the global search capability in the exploration phase and strengthening the local search capability in the development phase.
2. The Dimension Learning-Based Hunting (DLH) search strategy: By introducing the dimensional learning strategy, MIHHO can effectively balance global and local search capabilities and maintain the diversity of the population, thus enhancing the algorithm’s optimization ability.
3. Position update strategy based on Dung Beetle Optimizer (DBO) algorithm: A position update strategy based on the DBO algorithm is proposed, which reduces the possibility of the algorithm falling into a local optimal solution in the path planning process.
4. Performance test and analysis: The MIHHO algorithm significantly outperforms the other algorithms in terms of optimization-seeking ability, convergence speed, and stability through comparison experiments with 12 classical functions and the CEC2022 test function.
5. Path planning experiments: The performance of the MIHHO algorithm was comprehensively evaluated through global path planning simulation experiments in simple and complex environments using the optimal value, mean, and standard deviation of the path length and the number of iterations. The experimental results show that the improved strategy exhibits significant advantages in terms of the solution accuracy, convergence speed, and avoidance of local optima.

These contributions show that MIHHO has significant performance advantages in path planning tasks, providing shorter and more efficient path planning results than existing algorithms. Its effectiveness and applicability in different environments are demonstrated.

## 2. The Path Planning Optimization Problem

### 2.1. Environmental Modeling

The raster method is the most commonly used environment map modeling method in path planning, which is simple and effective and can significantly reduce the complexity of modeling the environment. Therefore, this paper adopts the raster method to construct the working environment model of the mobile robot. In the raster environment, the free area is represented by a value of 0, and the corresponding grid is filled with white color; the obstacle area is represented by a value of 1, and the corresponding grid is filled with black color. If there is a situation where an obstacle cannot occupy a grid, the obstacle is inflated to fill a grid. For the robot’s path planning problem, the search cannot cross the rectangular boundaries of the grid. In addition, it should be limited by obstacles, i.e., the robot’s trajectory cannot pass through a grid region where obstacles are present. The transformation relationship between the grid sequence number and the corresponding coordinates is shown in (1) [9]:

(1) $$\left\{ \begin{array}{l} x_{n}=\mathrm{mod}\left(n,R_{x}\right)-0.5\\ y_{n}=R_{y}+0.5-ceil\left(n/R_{y}\right) \end{array}\right.,$$

where $\left(x_{n},y_{n}\right)$ denotes the position coordinates of the nth grid, $x_{n}$ represents the horizontal and vertical coordinates of the nth grid, $y_{n}$ denotes the vertical coordinates of the nth grid, $R_{x}$ denotes the total number of rows of the environment model, $R_{y}$ denotes the total number of columns of the environment model, n is the serial number of the nth grid, and ceil() and mod() are the round-down function and remainder function, respectively.

### 2.2. Constraints and Fitness Functions

After setting up relevant environmental data, a population intelligence algorithm is used to find the ideal path in the map that satisfies all requirements. In addition, a fitness function that can contain constraints is created, and solutions that can satisfy this function are retained. Those that did not satisfy the fitness function were eliminated.

In MIHHO algorithm-based path planning, the position coordinates of the Harris hawk population are set to be updated at each iteration, representing a moving route for the robot. The MIHHO algorithm is used to find the optimal path from the start point to the goal point that meets the constraints from the 2D raster map.

1. Map boundary and obstacle constraints:

The robot’s moving path must be confined within the boundary of the raster map, and the constraints ub and lb are the boundaries of the search space for path planning. Any node $\left(x_{i},y_{i}\right)$ in the robot’s moving path must satisfy the following boundary conditions:

(2) $$\left\{ \begin{array}{l} lb_{x}\leq x_{i}\leq ub_{x}\\ lb_{y}\leq y_{i}\leq ub_{y} \end{array}\right.\begin{array}{cc} , & \forall i \end{array},$$

where $lb_{x}$ and $ub_{x}$ are the lower and upper limits of the horizontal boundary, and $lb_{y}$ and $ub_{y}$ are the lower and upper limits of the vertical boundary.

At the same time, the robot movement path cannot pass through the obstacle region, and any node $\left(x_{i},y_{i}\right)$ in the path must avoid the obstacle region $O_{j}$, that is,

(3) $$\left(x_{i},y_{i}\right)\notin O_{j}\begin{array}{cc} , & \forall i,j \end{array},$$

2. Path continuity conditions

The robot’s movement path in the access area should avoid overlapping paths and detours. Assuming that the position coordinate of the robot at the moment t is $\left(x_{t},y_{t}\right)$, the position coordinate of the robot at the next moment $\left(x_{t+1},y_{t+1}\right)$ should be satisfied:

(4) $$\begin{array}{ccc} x_{t+1}>x_{t} & or & y_{t+1}>y_{t} \end{array},$$

3. Path shortest condition

To realize mobile robot path planning, the robot must find the shortest path from the start to the goal point based on satisfying the boundary constraints and path continuity conditions. The length of the path is an important indicator of the merit of the path, and the goal of optimization is to minimize the total length of the path. The length of a path can be calculated by the Euclidean distance between all neighboring nodes on the path [28]. Specifically, the total length fit of a path can be expressed in the following form:

(5) $$fit=\sum_{i=1}^{m}\sqrt{{\left(x_{i+1}-x_{i}\right)}^{2}+{\left(y_{i+1}-y_{i}\right)}^{2}},$$

where $\left(x_{i},y_{i}\right)$ and $\left(x_{i+1},y_{i+1}\right)$ are the coordinates of two neighboring nodes on the path, and m is the total number of nodes on the path. The smaller the value of this objective function fit, the shorter the path is, and the merit of the path is thus evaluated. To achieve path optimization, the planning objective is to minimize the path length, which is

(6) min fit.

Eventually, by minimizing the path length, the optimal path that satisfies all the constraints can be found.

## 3. Harris Hawk Optimization Algorithm

The Harris Hawk Optimization (HHO) algorithm is a heuristic optimization algorithm based on Harris hawks’ behavior strategy. The algorithm realizes the global optimization of the optimization algorithm by imitating Harris hawks’ group hunting behavior and raid-hunting strategy. The hunting process is divided into the exploration stage, the transition from exploration to exploitation, and the exploitation stage [11].

### 3.1. Exploration Stage

As shown in [11], in the exploration stage, the individuals of the Harris hawk population randomly inhabit everywhere, track and detect prey according to their keen eyes, and search for prey globally according to Formula (7).

(7) $$X(t+1)=\left\{ \begin{array}{ll} X_{rand}(t)-r_{1}\left|X_{rand}(t)-2r_{2}X(t)\right|, & q\geq 0.5\\ \left[X_{rabbit}(t)-X_{m}(t)\right]-r_{3}\left[lb+r_{4}\left(ub-lb\right)\right], & q<0.5 \end{array}\right.,$$

where X(t) and X(t+1) are the position vectors of the individual at the current and next iteration, respectively; t is the number of iterations; $X_{rand}(t)$ is the randomly selected individual position; $X_{rabbit}(t)$ is the location of the rabbit; $r_{1}$, $r_{2}$, $r_{3}$, $r_{4}$, and q are random numbers generated from the segment (0,1); q is used to select the strategy to be adopted randomly; ub and lb are the upper and lower bounds of the search space; and $\left|X_{rand}(t)-2r_{2}X(t)\right|$ denotes a vector that takes absolute values element by element in a multidimensional space.

(8) $$\left|X_{rand}(t)-2r_{2}X(t)\right|=\left(\left|X_{rand,1}(t)-2r_{2}X_{1}(t)\right|,\left|X_{rand,2}(t)-2r_{2}X_{2}(t)\right|,\cdots ,\left|X_{rand,n}(t)-2r_{2}X_{n}(t)\right|\right)$$

$X_{m}(t)$ is the average position of the individual, and the expression is

(9) $$X_{m}(t)=\frac{\sum_{k=1}^{M}X_{k}(t)}{M},$$

where $X_{k}(t)$ is the k-th individual position in the population; M is the population size.

### 3.2. Transition from Exploration to Exploitation

The HHO algorithm transforms between exploration and different exploitation behaviors based on the escaped energy of the prey. The escaped energy is defined as follows:

(10) $$E=2E_{0}(1-\frac{t}{T}),$$

where $E_{0}$ is the initial energy of the prey, which is a random number from the segment (−1,1), and it is automatically updated at each iteration; t is the number of iterations; and T is the maximum number of iterations. When |E|≥1, it enters the exploration stage, and when |E|<1, it enters the exploitation stage.

### 3.3. Exploitation Stage

Based on the prey’s escape behavior and Harris hawks’ roundup strategy, combined with the prey’s escape energy E, the HHO algorithm proposes four strategies to simulate Harris hawks’ attack behavior during the exploitation stage. The probability of prey escaping is denoted by r. When r≥0.5, it means that the prey failed to escape; when r<0.5, the probability of successful prey escape is high.

(1) When 0.5≤|E|<1 and r≥0.5, the position update is soft besiege:

(11) $$X(t+1)=\Delta X(t)-E\left|JX_{rabbit}(t)-X(t)\right|,$$

where $\Delta X(t)=X_{rabbit}(t)-X(t)$ represents the difference between the position of the prey and the current position of the individual; J is a random number from the segment (0,2).

(2) When |E|<0.5 and r≥0.5, the position update is hard besiege:

(12) $$X(t+1)=X_{rabbit}(t)-E\left|\Delta X(t)\right|,$$

(3) When 0.5≤|E|<1 and r<0.5, the position update is soft besiege with progressive rapid dives:

(13) $$X(t+1)=\left\{ \begin{array}{l} Y,f(Y)<f(X(t))\\ Z,f(Z)<f(X(t)) \end{array}\right.,$$

(14) $$Y=X_{rabbit}(t)-E\left|JX_{rabbit}(t)-X(t)\right|,$$

(15) Z=Y+S×LF,

where f() is the fitness function; D is the dimension of the problem; S is a 1×D random vector; and LF is the Levy flight function, and its formula is

(16) $$LF=0.01\times \frac{\mu \times \delta }{{\left|\nu \right|}^{\frac{1}{\beta }}},$$

(17) $$\delta ={\left(\frac{\Gamma (1+\beta )\times \sin(\frac{\pi \beta }{2})}{\Gamma (\frac{1+\beta }{2})\times \beta \times 2^{\frac{\beta -1}{2}}}\right)}^{\frac{1}{\beta }},$$

where μ and ν are random values in (0,1); β is a random constant and is set to 1.5; and Γ is the standard gamma function with the following expression:

(18) $$\Gamma \left(x\right)=\int_{0}^{+\infty }e^{-t}t^{x-1}dt,$$

(4) When |E|<0.5 and r<0.5, the position update is hard besiege with progressive rapid dives:

(19) $$X(t+1)=\left\{ \begin{array}{l} Y,f(Y)<f(X(t))\\ Z,f(Z)<f(X(t)) \end{array}\right.,$$

(20) $$Y=X_{rabbit}(t)-E\left|JX_{rabbit}(t)-X_{m}(t)\right|,$$

(21) Z=Y+S×LF,

## 4. Proposed Algorithm

Due to some defects in the structure of HHO, the searchability is insufficient, the search process easily falls into local optima, and the convergence accuracy is low. Therefore, the HHO algorithm is improved by combining the double adaptive weights strategy, the Dimension Learning-Based Hunting (DLH) search strategy, and the position update strategy combined with the Dung Beetle Optimizer algorithm to improve its convergence speed and solution quality.

### 4.1. Double Adaptive Weights Strategy

The inertia weight factor plays an important role in HHO, which is closely related to the searchability of the algorithm. In the process of generating the Harris hawks’ position, the double adaptive weights $\omega_{1}$ and $\omega_{2}$ are used to update the Harris Hawks’ position. The weight values change with the iteration process and adapt to each other. The exploration ability and exploitation ability of HHO are improved, and the convergence accuracy and speed are improved. The specific formulas of $\omega_{1}$ and $\omega_{2}$ are as follows:

(22) $$\omega_{1}={\left(1-\frac{t}{T}\right)}^{1-\tan(\pi \cdot (rand-0.5))/T},$$

(23) $$\omega_{2}={\left(2-\frac{2t}{T}\right)}^{1-\tan(\pi \cdot (rand-0.5))/T},$$

The iterative process is divided into two stages. In the exploration stage, Equation (7) is updated as follows:

(24) $$X(t+1)=\left\{ \begin{array}{ll} \omega_{1}X_{rand}(t)-r_{1}\left|X_{rand}(t)-2r_{2}X(t)\right|, & q\geq 0.5\\ \omega_{1}\left[X_{rabbit}(t)-X_{m}(t)\right]-r_{3}\left[lb+r_{4}\left(ub-lb\right)\right], & q<0.5 \end{array}\right.,$$

In the exploitation stage, Equations (11), (12), (14) and (20) are updated as follows:

(25) $$X(t+1)=\omega_{2}\Delta X(t)-E\left|JX_{rabbit}(t)-X(t)\right|,$$

(26) $$X(t+1)=\omega_{2}X_{rabbit}(t)-E\left|\Delta X(t)\right|,$$

(27) $$Y=\omega_{2}X_{rabbit}(t)-E\left|JX_{rabbit}(t)-X(t)\right|,$$

(28) $$Y=\omega_{2}X_{rabbit}(t)-E\left|JX_{rabbit}(t)-X_{m}(t)\right|,$$

After generating the new position, it is checked whether it falls within the upper and lower bounds of the population search space. If it is not satisfied, it is adjusted according to Equation (29) so that it falls within the feasible solution space.

(29) X(t+1)=max(min(X(t+1),ub),lb),

### 4.2. Dimension Learning-Based Hunting (DLH) Search Strategy

Population diversity plays a key role in the convergence speed and accuracy of the algorithm. The population diversity of HHO gradually decreases with the iteration of the algorithm, which leads to the solution of high-dimensional complex problems such as local optima, and the global optimal solution cannot be found. Therefore, to ensure that the population maintains a rich diversity in the iteration, the Dimension Learning-Based Hunting (DLH) search strategy is used. DLH uses different methods to construct a neighbor for each Harris hawk that can share information. This dimensional learning method can improve the ability of Harris hawks to balance exploration and exploitation and maintain diversity [29].

As shown in [29], in the DLH search strategy, each new position of the Harris hawk $X_{i}(t)$ is calculated using Equation (32), and the individual hawk is learned by its different neighbors and randomly selected Harris hawks in the population. In addition to randomly generated locations through initialization and locations that are continually updated during the iteration process based on the exploration and development position $X_{i}(t+1)$, a new candidate Harris hawk $X_{id}(t+1)$ is generated for the new position of Harris hawk $X_{i}(t)$ in the DLH search strategy. To this end, the Euclidean distance between the current position $X_{i}(t)$ and its candidate position $X_{i}(t+1)$ is calculated to obtain $R_{i}(t)$, and the formula is as follows:

(30) $$R_{i}(t)=\left\|X_{i}(t)-X_{i}(t+1)\right\|,$$

Then, the neighbor $N_{i}(t)$ of $X_{i}(t)$ is constructed by Equation (31) for $R_{i}(t)$:

(31) $$N_{i}(t)=\left\{X_{j}(t)|D_{i}(X_{i}(t),X_{j}(t))\leq R_{i}(t),X_{j}(t)\in Pop\right\},$$

The constructed neighborhood $X_{i}(t)$ is learned by multi-neighborhood learning of Equation (32), where $X_{id}(t+1)$ the d-th dimension is calculated using the random neighbor $X_{n,d}(t)$ of the d-th dimension selected from $N_{i}(t)$ and the random Harris Hawk $X_{r,d}(t)$ selected from the population as follows:

(32) $$X_{id}(t+1)=X_{i,d}(t)+rand\times (X_{n,d}(t)-X_{r,d}(t)),$$

Therefore, in the selection and update phase, the optimal candidate is selected by comparing the fitness values of the two candidate solutions through Equation (33):

(33) $$X_{i1}(t+1)=\left\{ \begin{array}{l} X_{i}(t+1),f(X_{i})<f(X_{ig})\\ X_{id}(t+1),f(X_{i})\geq f(X_{ig}) \end{array}\right.,$$

The selected candidate fitness value is updated if it is less than $X_{i}(t+1)$, otherwise it remains unchanged.

### 4.3. Position Update Strategy Based on Dung Beetle Optimizer Algorithm

The Dung Beetle Optimizer (DBO) algorithm is a new swarm intelligence optimization algorithm proposed by Xue Jiankai et al. in 2022 [30], which simulates the rolling, dancing, foraging, stealing, and breeding behaviors of dung beetles. The algorithm takes into account both global exploration and local development, so it has the characteristics of a fast convergence speed and high accuracy and can effectively solve complex optimization problems. According to the idea that the rolling ball cockroach uses the sun’s rays to navigate in the DBO, the dung ball is rolled in a straight line, and a new position is introduced into the HHO. It enables individuals in the population to choose the most suitable direction, to avoid falling into the local minimum prematurely.

As shown in [30], the location update method of the ball-rolling cockroach is as follows:

(34) $$\begin{array}{l} X_{i-DBO}(t+1)=X_{i}(t)+\alpha \times k\times X_{i}(t-1)+b\times \Delta x\\ \;\Delta x=\left|X_{i}(t)-X^{w}\right| \end{array},$$

where $X_{i}(t)$ denotes the position of the i-th cockroach at the t-th iteration; k∈(0,0.2] denotes the deflection coefficient; b is a fixed value from the segment (0,1); and α is the position deviation of the dung beetle due to the influence of natural factors, which is taken as a fixed value of −1 or 1. When the value is 1, it means there is no deviation, and the value of −1 represents the deviation direction; $X^{w}$ is the worst position in the global; and Δx is the variation in light intensity. The larger the value is, the weaker the light source is, which promotes the dung beetle to avoid this position. In this way, the whole search space in the optimization process is explored as thoroughly as possible, and the possibility of falling into the local optimum is reduced.

Then, the fitness values of the two candidate positions are compared to select the better candidate positions.

(35) $$X_{i}(t+1)=\left\{ \begin{array}{l} X_{i-DBO}(t+1),f(X_{i-DBO})<f(X_{i1})\\ X_{i1}(t+1),f(X_{i-DBO})\geq f(X_{i1}) \end{array}\right.,$$

### 4.4. MIHHO Algorithm Flow

**Figure 1:**
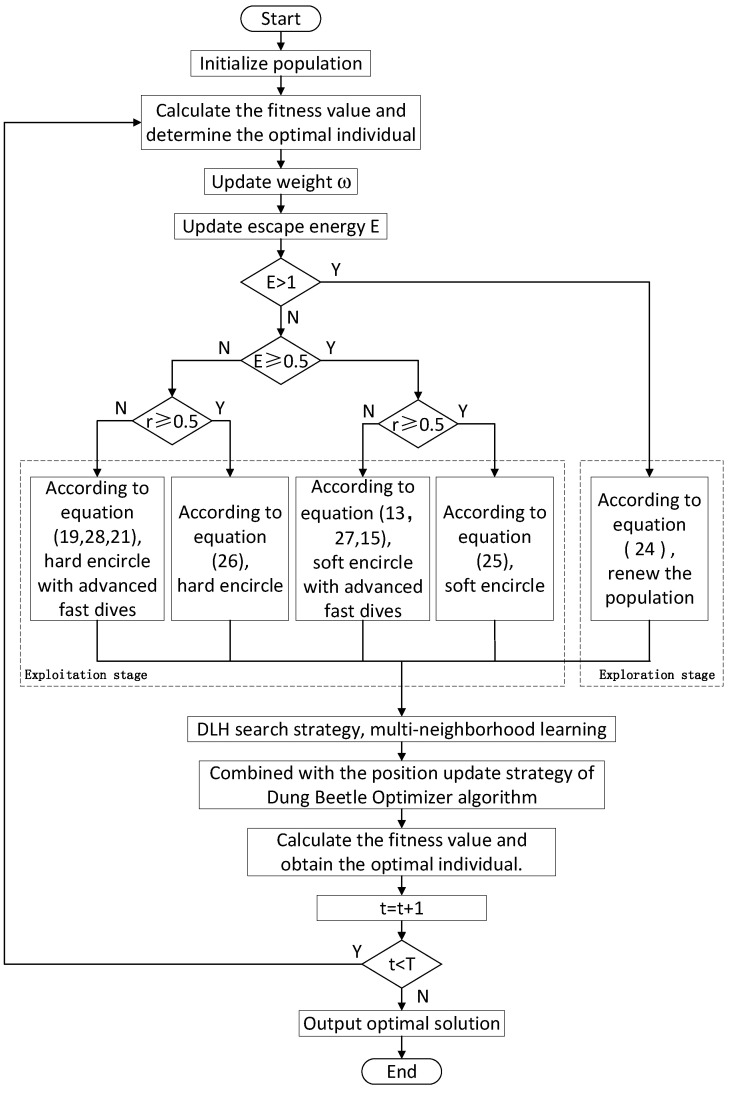
MIHHO algorithm flow chart.

In summary, this paper improves the HHO algorithm, introduces three strategies, and proposes the MIHHO. The algorithm flow is as follows, and the flow chart is shown in Figure 1:

- Step 1: The basic parameters of the Harris hawk population are initialized, including the number of populations N, the maximum number of iterations of the algorithm T, and the starting position of all individuals in the solution space.
- Step 2: The iteration starts, the fitness value of all individuals in the population is obtained, and the best individual is obtained.
- Step 3: The double adaptive weight strategy (Equations (22) and (23)) is used to dynamically adjust the position of the Harris hawk to improve the search efficiency.
- Step 4: The Harris hawk population updates its position through different strategies. In the exploration phase, two strategies are used to detect prey based on the q-value (Equation (24)). In the exploitation phase, prey are attacked using different strategies based on their escape energy and escape probability, including soft besiege (Equation (25)), hard besiege (Equation (26)), soft besiege with progressive rapid dives (Equations (13), (15) and (27)), and hard besiege with progressive rapid dives (Equations (19), (21) and (28)).
- Step 5: Multi-neighborhood learning is performed according to the DLH search strategy (Equations (30)–(33)).
- Step 6: The fitness value of all individuals in the population is calculated, and the best and worst individuals are obtained.
- Step 7: According to the DBO’s solar ray navigation strategy (Equations (34) and (35)), the positions of individuals caught in localized extremes are updated.
- Step 8: The optimal individual position and fitness value so far are calculated.
- Step 9: The updated new population is returned, and it is determined whether the algorithm terminates. If there is no termination, the second step is followed to continue the execution.
- Step 10: The condition is terminated and the final optimal individual of the population and its fitness value are output.

### 4.5. Time Complexity Analysis

A time complexity analysis usually depends on three processes: population initialization, the calculation of the fitness function, and population location update. It is related to the population size N, the search space dimension D, and the maximum number of iterations T. For HHO, the total computational complexity is O(N)+O(T×N)+O(T×N×D), that is, O(N×(T+T×D+1)). For the MIHHO proposed in this paper, although the double adaptive weight strategy, the DLH search strategy, and the position update strategy of the DBO are introduced, these improved strategies do not increase significantly in computational complexity. The improvement strategy is still carried out within the original framework without the additional complexity of the algorithm. Therefore, the total computational complexity of MIHHO is still O(N×(T+T×D+1)).

The analysis shows that the computational complexity of HHO is consistent with that of MIHHO, and the improved strategy does not increase the additional time complexity.

## 5. MIHHO Performance Test and Analysis

### 5.1. Solving the Classical Functions

The experimental simulation platform used was a Windows 10 computer, the model used was 12 GB RAM, Intel (R) Core (TM) i5-10210 U CPU, and the software used was MATLAB R2021b. To verify the performance of the MIHHO algorithm, the Grey Wolf Optimizer (GWO) [31], the Sparrow Search Algorithm (SSA) [32], the Whale Optimization Algorithm (WOA) [33], the Particle Swarm Optimization (PSO) [34], the Gradient-Based Optimizer (GBO) [35], Sand Cat Swarm Optimization (SCSO) [36], the Dung Beetle Optimizer (DBO) [30], and Harris Hawk Optimization (HHO) [11] were selected to compare with the MIHHO algorithm proposed in this paper. To ensure the fairness and effectiveness of the experiment, the population size and the number of iterations of all algorithms were set to 30 and 500, and other internal parameters of each algorithm were consistent with the settings in the original literature.

In order to test the effectiveness of the MIHHO improvements, tests were performed in the classical functions [37]. Twelve classical functions with different characteristics were used for testing. Table 1 shows information about the 12 classical functions. Among them, F1–F4 are single-peak test functions to test the development ability of the algorithms, and F5–F12 are 30-dimensional and fixed-dimensional multi-peak test functions to test the exploration ability of the algorithms and their ability to avoid local optima.

To eliminate the influence of randomness on the test results, each algorithm was run independently for each test function 30 times, and the mean, standard deviation, and optimal value were used as the algorithm performance evaluation index. The smaller the mean value, the stronger the convergence accuracy and optimization ability of the algorithm; the smaller the standard deviation, the smaller the fluctuation in the algorithm and the higher the stability. The comparison algorithm was tested with the 12 classical functions given in Table 1, and the results are shown in Table 2, where mean denotes the average rank on the accuracy of all function findings, and rank denotes the ranking based on the mean. It can be seen that the MIHHO algorithm shows excellent optimization ability and ranks first. On F1, F2, F3, F6, and F8, MIHHO reaches the optimal value, and on F9, F10, and F12, although it does not reach the theoretical optimal value, it is the closest to the theoretical optimal value, which shows its strong optimization ability. In addition, MIHHO performs stably on the mean and standard deviation, indicating that its results remain consistent over many experiments, especially on F1, F2, F3, F6, and F8. In function F5, although MIHHO reaches the same optimal value as DBO, HHO, and WOA, the standard deviation of MIHHO is several tens of orders of magnitude lower than the other algorithms.

**Figure 2:**
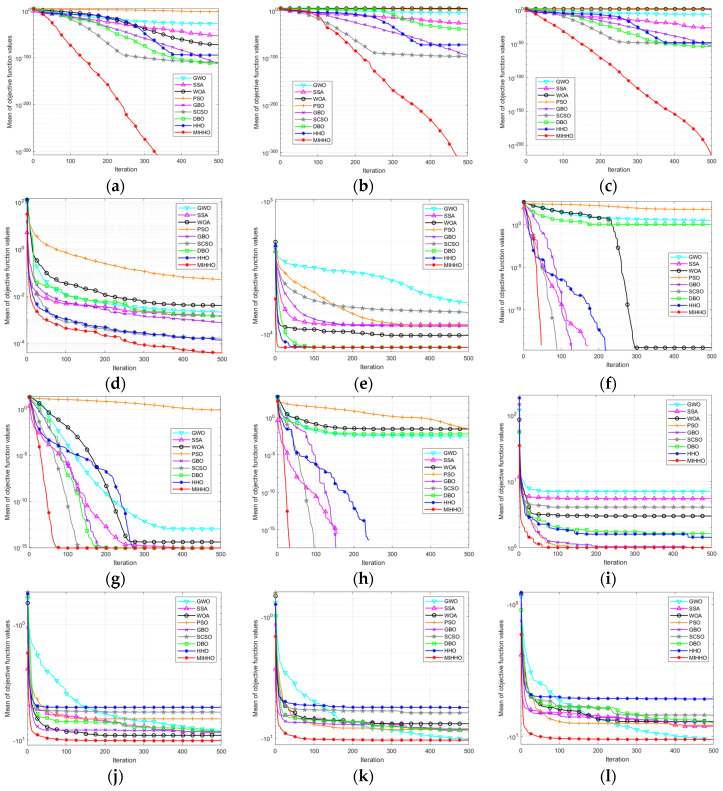
Classical functions convergence curves. (**a**) F1 convergence curve; (**b**) F2 convergence curve; (**c**) F3 convergence curve; (**d**) F4 convergence curve; (**e**) F5 convergence curve; (**f**) F6 convergence curve; (**g**) F7 convergence curve; (**h**) F8 convergence curve; (**i**) F9 convergence curve; (**j**) F10 convergence curve; (**k**) F11 convergence curve; (**l**) F12 convergence curve.

Figure 2 shows the average iteration curves of the different algorithms after 30 runs. Based on the analysis of the convergence curves, MIHHO performs well on most of the tested functions. First, in functions F1, F2, F3, F6, F7, F8, and F12, MIHHO demonstrates the fastest convergence speed, and its convergence process is nearly linear and fast approaching the theoretical optimum, which indicates that the algorithm has a strong ability to jump out of the local optimum. In addition, the curves of MIHHO are usually lower than those of other algorithms, which indicates that MIHHO achieves better optimization results with the same number of iterations.

The Wilcoxon rank sum test [38] is a nonparametric statistical test that can detect more complex data distributions. The significance level is set to 5%, and when the *p*-value is less than 0.05, there is considered to be a significant difference between the two algorithms, and when *p* is greater than 0.05, this indicates that the difference between the two algorithms is not obvious, and the emergence of “NAN” indicates that the two comparative algorithms obtain similar results of optimization and cannot be verified as a significant difference. In this paper, the Wilcoxon rank sum test is performed on the best results of 30 independent runs of MIHHO and eight comparison algorithms to demonstrate the significant difference between the algorithms. According to the magnitude of the *p*-value, the symbols “+/=/−” are used to indicate that the comparison algorithm is significantly different from MIHHO, that a significant difference could not be verified, and that the difference is not significant, respectively. From the results given in Table 3, it can be seen that the *p*-values of the Wilcoxon rank sum test results for MIHHO are overwhelmingly less than 5%, except for the almost identical performance of the algorithms’ optimization search on F6, F7, and F8, which indicates that MIHHO has a significant difference over the other algorithms.

**Figure 3:**
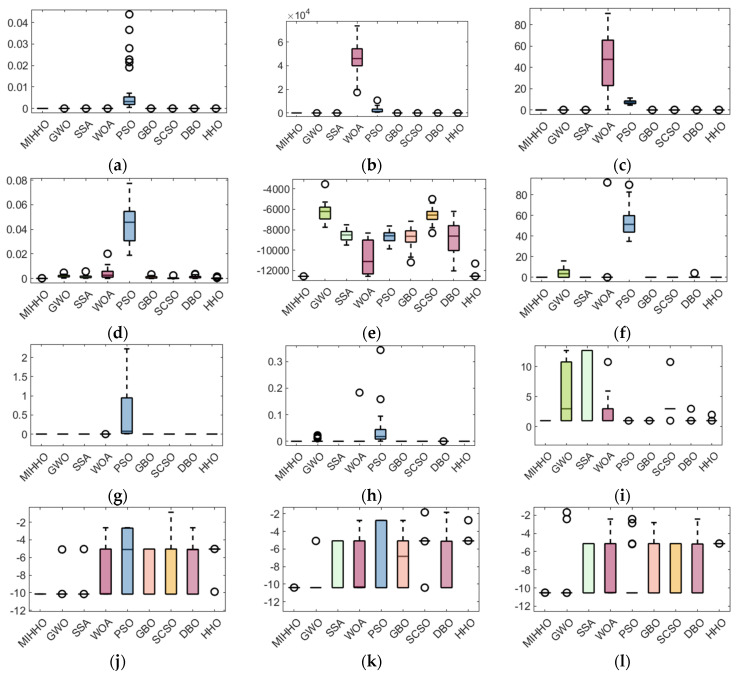
Boxplots of MIHHO algorithm with other comparative algorithms. (**a**) F1 test function; (**b**) F2 test function; (**c**) F3 test function; (**d**) F4 test function; (**e**) F5 test function; (**f**) F6 test function; (**g**) F7 test function; (**h**) F8 test function; (**i**) F9 test function; (**j**) F10 test function; (**k**) F11 test function; (**l**) F12 test function.

A box plot is often used to visualize the distribution of data [39], and in this paper, the stability of the algorithms is verified through box plots. Figure 3 demonstrates the distribution of the optimal fitness values of the comparison algorithms after 30 experiments. From Figure 3, it can be seen that for functions F1, F2, F3, F6, F7, F8, F9, and F10, MIHHO has the lowest box height, and compared with the other algorithms, the data of MIHHO are more concentrated and there are no anomalies, while the other algorithms have anomalies. This indicates that MIHHO shows higher stability in the optimization search process with less data fluctuation, which is significantly better than the other algorithms.

In summary, compared with the rest of the algorithms, MIHHO exhibits a higher convergence speed and better exploration and exploitation ability, and it can effectively avoid falling into local optima, proving that the improvement strategy proposed in this paper improves the comprehensive performance of the algorithm.

### 5.2. Solving the CEC2022 Test Functions

To further verify the algorithm’s optimization performance and robustness on complex test functions, the CEC2022 test function is selected for the optimization comparison analysis of six algorithms [40]. Table 4 lists detailed information about functions F13–F24 (UF is a Unimodal Function, BF is a Basic Function, HF is a Hybrid Function, and CF is a Composition Function).

Each algorithm was run independently 30 times with a population size of 30, an optimization problem dimension of 10, and 1000 iterations, and the other internal parameters of each algorithm were kept consistent with their settings in the original literature; the results of the optimization search are shown in Table 5. As can be seen from Table 5, the MIHHO algorithm has the second-highest performance among the CEC2022 test functions after GBO. However, for the unimodal function F13, MIHHO has the lowest mean and standard deviation, whereas the performance of the HHO with WOA optimization search is poor. On the basic functions, MIHHO is very close to the theoretical value even though it does not converge to the theoretical optimum every time, and it has a large improvement compared to HHO. On the hybrid functions F18 and F20, MIHHO’s average value is better than those of the other algorithms, showing excellent overall performance. On the composition functions, the convergence accuracy of MIHHO is still ahead of that of the other algorithms, and the algorithm optimization is more stable. In summary, MIHHO has a greater advantage over the other algorithms in terms of optimization performance, generally higher robustness, and the ability to solve complex optimization problems, showing that the improvement strategy can effectively overcome the defects of HHO.

**Figure 4:**
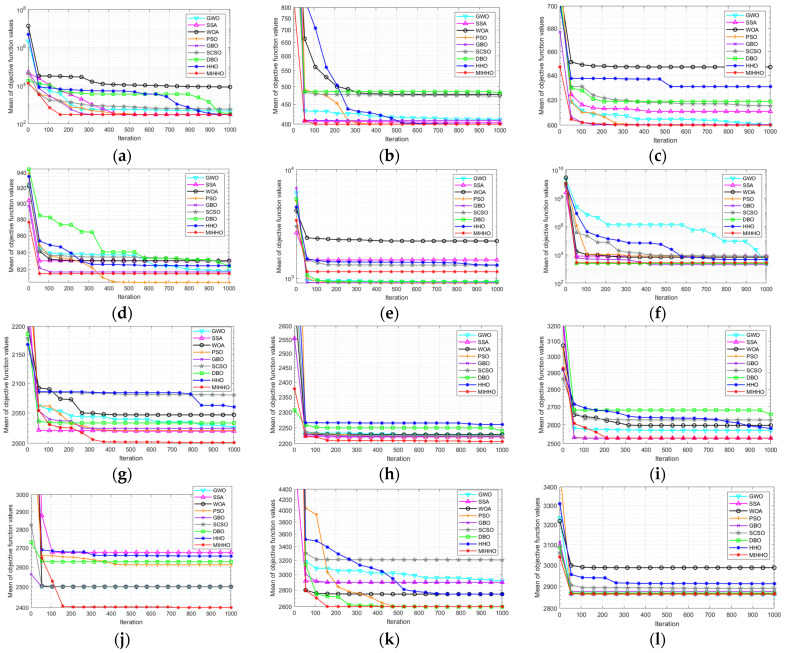
CEC2022 functions’ convergence curves. (**a**) F13 convergence curve; (**b**) F14 convergence curve; (**c**) F15 convergence curve; (**d**) F16 convergence curve; (**e**) F17 convergence curve; (**f**) F18 convergence curve; (**g**) F19 convergence curve; (**h**) F20 convergence curve; (**i**) F21 convergence curve; (**j**) F22 convergence curve; (**k**) F23 convergence curve; (**l**) F24 convergence curve.

Figure 4 shows the average iteration curves of different algorithms after 30 runs. As can be seen from Figure 4, on the unimodal function F13, MIHHO achieves a better optimization result with the same number of iterations. On the basic functions F16 and F17, MIHHO does not have a clear advantage but still converges to the optimal value at a speed not inferior to the other algorithms. For the hybrid functions, although MIHHO does not converge to the optimal point, MIHHO’s convergence accuracy is stronger than that of the other algorithms, and the speed of jumping out of the local optimum is also faster. For composition functions, MIHHO not only converges to the optimum quickly, but also has higher accuracy than other algorithms, indicating that MIHHO has a strong ability to jump out of the local optimum.

Table 6 shows the Wilcoxon rank sum test results of MIHHO with other algorithms on the CEC2022 test functions. As can be seen in Table 6, MIHHO performs “12/0/0” in comparison with GWO and SCSO, and in all 12 functions, MIHHO’s performance is significantly different from these two algorithms. As can be seen in Table 3, the *p*-values of most of the 12 functions tested are less than 0.05, indicating that MIHHO is significantly different than the other algorithms.

**Figure 5:**
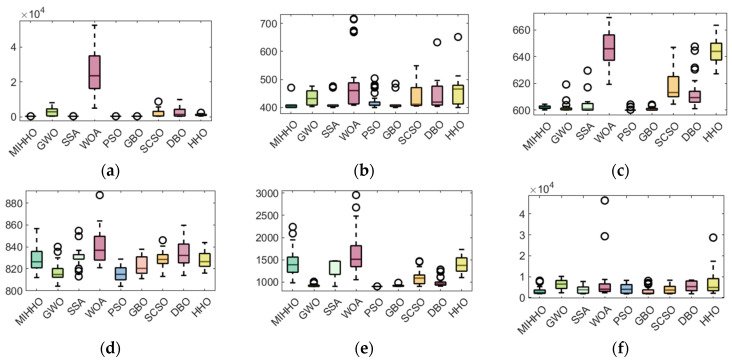

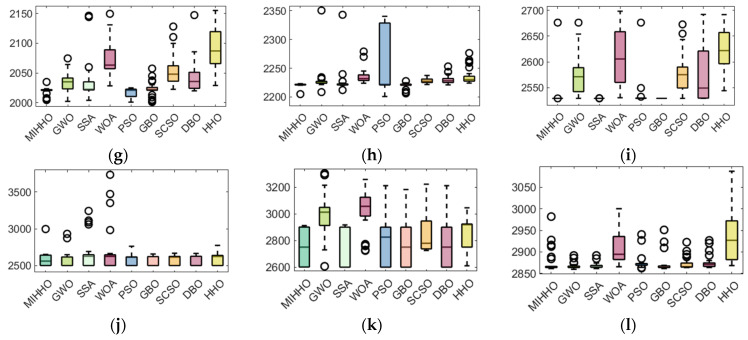
Boxplot of MIHHO algorithm compared with other algorithms. (**a**) F13 test function; (**b**) F14 test function; (**c**) F15 test function; (**d**) F16 test function; (**e**) F17 test function; (**f**) F18 test function; (**g**) F19 test function; (**h**) F20 test function; (**i**) F21 test function; (**j**) F22 test function; (**k**) F23 test function; (**l**) F24 test function.

As shown in Figure 5, MIHHO’s boxplots on the CEC2022 test functions demonstrate its excellent performance stability and significant advantages. Specifically, on functions F13, F14, and F15, MIHHO’s boxplots show a high degree of stability, indicating that its performance fluctuates very little for these functions. For functions F20, F21, and F24, MIHHO can find even smaller values, further validating its strong ability to optimize these functions. Overall, MIHHO’s performance on the CEC2022 test function set demonstrates its excellent performance stability when dealing with complex optimization problems and its clear advantages on specific function types.

In summary, MIHHO performs well in the CEC2022 test, showing a faster convergence speed and stronger exploration and exploitation ability, and it can effectively avoid falling into local optima. The experimental results prove that the improvement strategy proposed in this paper significantly improves the comprehensive performance of the algorithm.

### 5.3. Comparison with Other Authors Improving HHO

**Figure 6:**
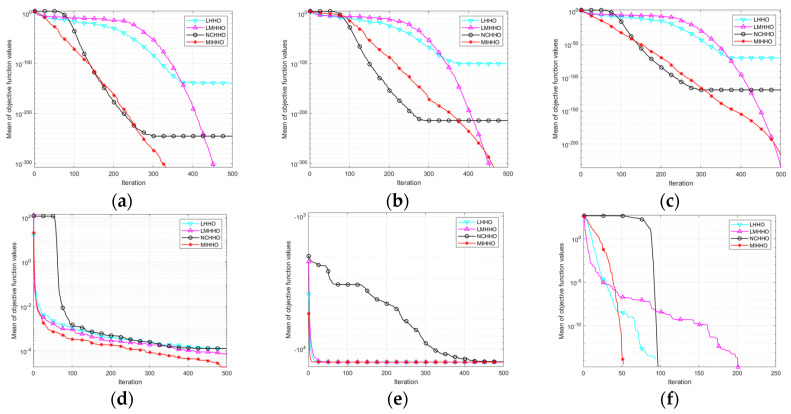

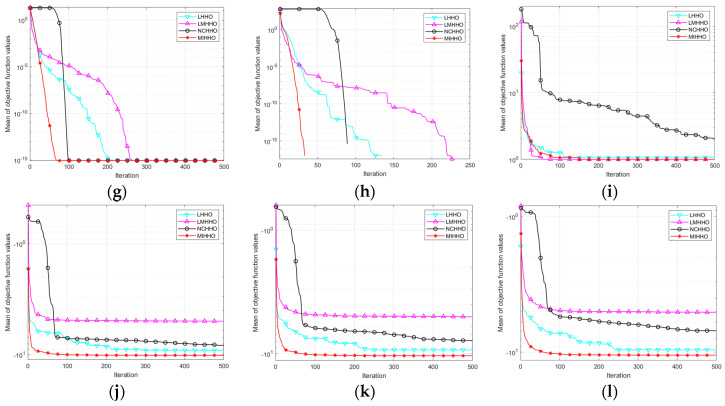
Function convergence curves. (**a**) F1 convergence curve; (**b**) F2 convergence curve; (**c**) F3 convergence curve; (**d**) F4 convergence curve; (**e**) F5 convergence curve; (**f**) F6 convergence curve; (**g**) F7 convergence curve; (**h**) F8 convergence curve; (**i**) F9 convergence curve; (**j**) F10 convergence curve; (**k**) F11 convergence curve; (**l**) F12 convergence curve.

The above experiments were compared with the superior algorithms of recent years, and better results were obtained. In this section, using the classical functions mentioned in Section 5.1, MIHHO is compared with the HHO algorithms that have been improved by other authors, namely Nonlinear-based Chaotic Harris Hawks Optimization (NCHHO) [27], Leader Harris Hawk Optimization (LHHO) [41], and Long-Term Memory Harris Hawk Optimization (LMHHO) [42], and the results of the experiments are shown in Table 7 and Figure 6.

In the convergence curve, MIHHO is significantly better than the other three improved algorithms; regarding the convergence speed in the first and middle stages or the convergence accuracy at the end, MIHHO is in first place. In terms of specific data, except for F6, F7, and F8, where the algorithms have the same optimization results, all of the statistical values in the other functions significantly outperform the optimization results of the other improved algorithms, indicating that the improvement strategy used by MIHHO is superior. For the unimodal functions, the chaotic and nonlinear control parameters used by NCHHO speed up the convergence of the algorithm to some extent, but no global optimum is found. From Table 7 and Figure 6, it is clear that MIHHO is much better at dealing with complex problems, and the results also reaffirm the validity of MIHHO’s ability to balance exploration and development, convergence speed, accuracy, and other properties.

### 5.4. Ablation Experiment

To analyze the impact of different strategies on the performance of the algorithm, three strategies of MIHHO are compared through experiments in this section. Table 8 shows the specific details of the strategies involved in the comparison. In Table 8, “0” indicates that the strategy is not introduced into the algorithm, while “1” indicates that the strategy is introduced. Some of the test functions are selected for testing, while other parameters are the same as before, and the results of the optimization search are shown in Table 9.

**Figure 7:**
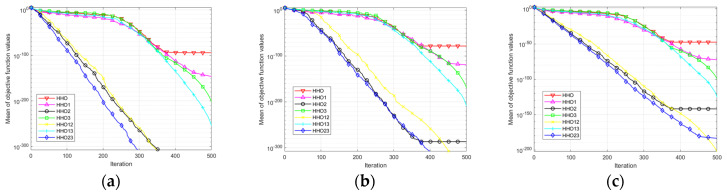

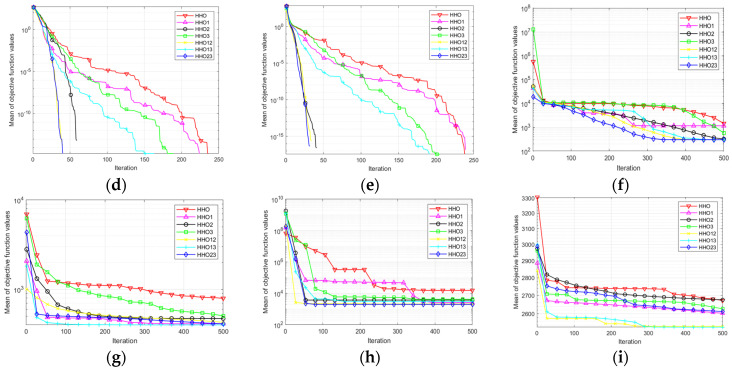
Convergence curves of ablation experiments. (**a**) F1 convergence curve; (**b**) F2 convergence curve; (**c**) F3 convergence curve; (**d**) F6 convergence curve; (**e**) F8 convergence curve; (**f**) F13 convergence curve; (**g**) F14 convergence curve; (**h**) F18 convergence curve; (**i**) F21 convergence curve.

As can be seen in Figure 7 and Table 9, the three improved strategies have different degrees of improvement in HHO in terms of the search accuracy and convergence speed. In the unimodal functions, the DLH search strategy performs best. In functions F6 and F8, the original HHO algorithm can reach the global optimum, but the algorithm converges faster after adding the improved strategies. In function F13, although strategy 1 and strategy 3 do not significantly improve the hunting accuracy of the HHO algorithm, the combination of strategies 1 and 3 (HHO13) converges to an approximate theoretical optimum. In summary, all three improvement strategies have a positive influence on the original algorithm, and the combination of the three algorithms optimizes the performance improvement of the algorithm, proving the effectiveness of the improvement strategies.

## 6. The Application of MIHHO in Path Planning

In this study, several experiments were designed with the aim of demonstrating the advantages of MIHHO in terms of the solution accuracy, convergence speed, and avoidance of becoming stuck in local optimization. The experiments cover both simple and complex environments and are compared with a variety of algorithms to fully evaluate the performance of MIHHO.

### 6.1. Steps in the Path Planning Experiment

The position coordinates of the Harris hawk population are updated at each iteration to represent a moving route for the robot. The MIHHO algorithm is used to find the optimal path from the start point to the goal point that meets the constraints of the 2D raster map. The experimental steps are as follows:

- Step 1: Set the initialization parameters. Set the robot start position S, goal position G, population number N, and iteration number T, and set the map boundary conditions and obstacle positions.
- Step 2: Initialize the population. Randomly generate N paths from the starting point to the endpoint and detect whether they are within the map boundary.
- Step 3: Calculate the degree of adaptation. Evaluate the quality of each path using the objective function (Equation (5)) to determine the best path in the initialization.
- Step 4: Update the weights according to the double adaptive weight formula (Equations (22) and (23)) to improve the probability of finding the optimal path.
- Step 5: In the HHO for path optimization, two strategies are used in the exploration phase to detect the prey through the q-value (Equation (24)). In the exploitation phase, based on the prey’s escape energy and escape probability, different strategies are selected for attacking, including soft besiege (Equation (25)), hard besiege (Equation (26)), soft besiege with progressive rapid dives (Equations (13), (15) and (27)), and hard besiege with progressive rapid dives (Equations (19), (21) and (28)).
- Step 6: Perform multi-neighborhood learning according to the DLH search strategy (Equations (30)–(33)). By searching in different dimensions, the algorithm can find several different paths, increasing the likelihood of finding a better path.
- Step 7: Calculate the fitness values of all individuals of the population and obtain the optimal and worst paths.
- Step 8: Position update strategy based on DBO algorithm (Equations (34) and (35)), update the position of an individual caught in a local extreme to help it jump out of the local optimum and continue searching for a better solution.
- Step 9: Calculate the optimal path and fitness value so far.
- Step 10: Return the updated new population and determine whether the algorithm termination condition is reached; if not, then jump to the second step to continue execution.
- Step 11: Terminate the condition and output the final optimal path of the obtained population and its fitness value.

### 6.2. Analysis of Path Planning Experimental Results 

The environments are categorized into two types, namely simple and complex environments, according to the proportion of obstacles in the environment [43]. Simple and complex environments include two sizes, 20 × 20 and 40 × 40, respectively. Compared with complex environments, simple environments have fewer obstacles with regular shapes and sparse distribution, and these obstacles are easy to recognize and bypass and do not cause serious problems in path planning and task execution. Complex environments have a higher proportion of obstacles with irregular shapes and dense distribution, which pose a greater challenge to path planning algorithms. With this categorization method, the performance of different algorithms in various environments can be effectively evaluated.

To verify the performance of MIHHO in path planning, GWO, SSA, WOA, PSO, GBO, SCSO, DBO, and HHO were chosen as the comparison algorithms to conduct path planning experiments with MIHHO in the same raster map. To ensure the fairness and validity of the experiments, the population size and iteration number of all algorithms were set to 50 and 100, and the other internal parameters of each algorithm were kept consistent with their settings in the original literature.

#### 6.2.1. Experimental Simulation in a Simple Environment

The individual algorithms were tested in a simple environment on maps of two sizes, 20 × 20 and 40 × 40. The percentage of obstacles was set to 20% and these obstacles were regularly distributed and regular in shape. The test site was relatively empty, and this setup ensured that the obstacles were easy to bypass, which made path planning and task execution smoother, and thus allowed for a more effective evaluation of the basic performance of each algorithm in a simple environment.

**Figure 8:**
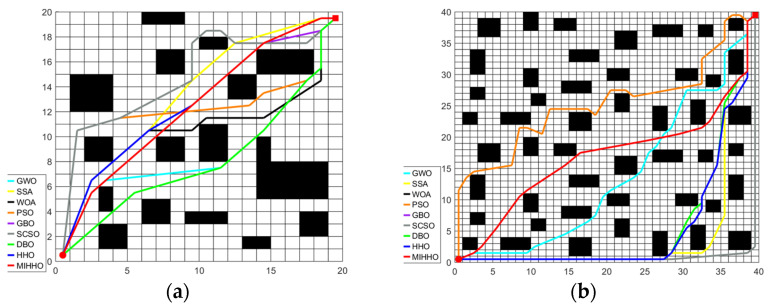
The simulation results of path planning in simple environments. (**a**) The simulation results of path planning in a 20 × 20 grid map in a simple environment. (**b**) The simulation results of path planning in a 40 × 40 grid map in a simple environment.

**Figure 9:**
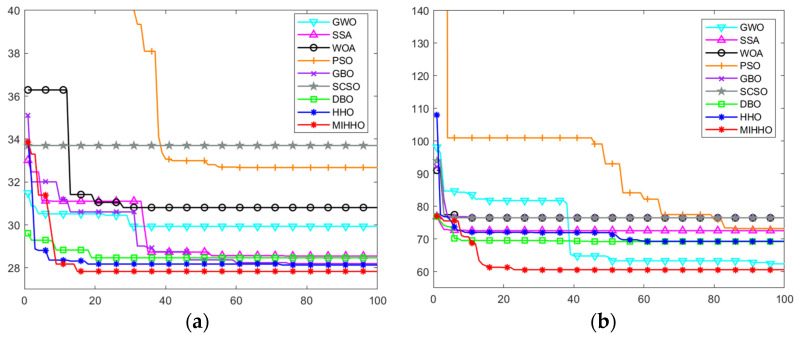
Convergence curves for path planning in simple environments. (**a**) The convergence curves of each algorithm in a 20 × 20 grid map in a simple environment. (**b**) The convergence curves of each algorithm in a 40 × 40 grid map in a simple environment.

Figure 8 shows the path planning simulation results of each algorithm for different sizes (20 × 20 and 40 × 40) in the simple environment, while Figure 9 shows the path planning convergence curves of each algorithm in this environment. It can be seen that the MIHHO algorithm can plan smoother paths in a simple environment compared to the other algorithms, which indicates that the MIHHO algorithm is more efficient for path planning. The PSO algorithm, on the other hand, produces more inflection points and detours in this environment, indicating that it may be more likely to fall into a local optimum during path planning. In Figure 9, the MIHHO algorithm converges faster and finds shorter paths in fewer iterations. In contrast, other algorithms such as GWO and PSO converge relatively slowly and show large fluctuations during the iterations.

Considering the influence of chance factors on the experimental results, we carried out several experimental simulations, and each algorithm was run independently 10 times on two kinds of maps to determine the optimal value, average value, and standard deviation of the path length and the optimal value, average value, and standard deviation of the number of iterations, and we comprehensively evaluated the algorithms’ optimization results in the path planning problem. The algorithms’ performance is compared in Table 10.

As can be seen in Table 10, the MIHHO algorithm successfully converges to the optimal solution after 18 iterations in a 20 × 20 raster map with an average path length of 28.35375. The path length of MIHHO is reduced by 2.23% compared to GWO, 3.29% compared to SSA, 10.93% compared to WOA, 12.08% compared to PSO, 4.66% compared to GBO, 14.11% compared to SCSO, 1.98% compared to DBO, and 1.99% compared to HHO. These results show that MIHHO can plan shorter paths in simple environments, demonstrating excellent path optimization capabilities. In addition, MIHHO is more stable in path planning with less fluctuation in path length and can converge to the optimal solution faster with significantly improved computational efficiency.

In the 40 × 40 raster map, although the SCSO algorithm has the lowest average number of iterations, it has a longer path length and is not as well optimized as MIHHO. MIHHO has an average path length of 61.05562, which is 17.59% less than GWO, 14% less than SSA, 18.92% less than WOA, 45.86% less than PSO, 13.04% less than GBO, 19.63% less than SCSO, 9.22% less than DBO, and 14.45% less than HHO. These results show that MIHHO can still maintain a short path length when dealing with larger scale path planning problems, showing its stability in large-scale path planning.

Therefore, compared to the other eight algorithms, the MIHHO algorithm has a shorter average path length and a lower average number of iterations and presents some advantages in the overall search performance and convergence speed.

#### 6.2.2. Experimental Simulation in Complex Environments

**Figure 10:**
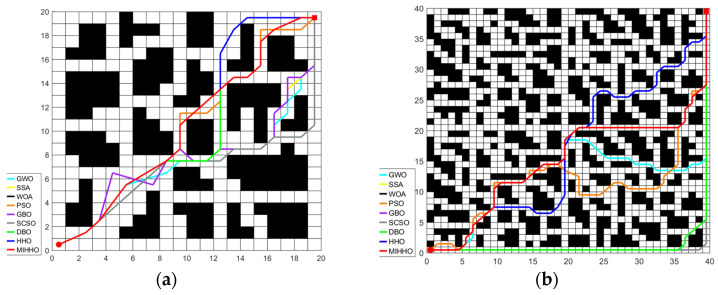
The simulation results of path planning in complex environments. (**a**) The simulation results of path planning in a 20 × 20 grid map in a complex environment. (**b**) The simulation results of path planning in a 40 × 40 grid map in a complex environment.

**Figure 11:**
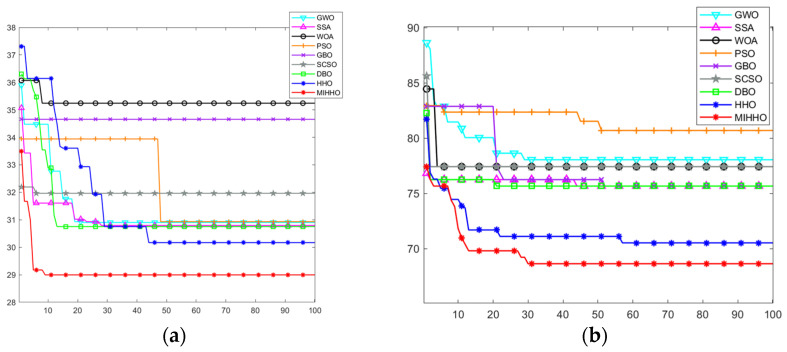
Convergence curves for path planning in complex environments. (**a**) The convergence curves of each algorithm in a 20 × 20 grid map in a complex environment. (**b**) The convergence curves of each algorithm in a 40 × 40 grid map in a complex environment.

In the complex environment, each algorithm was tested on maps of two sizes, 20 × 20 and 40 × 40. Obstacles occupy 40% of the map, with random distribution and different shapes, resulting in highly complex and crowded maps. This setup increases the difficulty of detouring and makes path planning and task execution more challenging, allowing for a more comprehensive evaluation of the performance of each algorithm in complex environments. Figure 10 and Figure 11 show the simulation results of path planning.

The MIHHO algorithm shows significant advantages in complex environments with 40% obstacles. Its path planning results show smoother and more direct paths, indicating that MIHHO has superior obstacle avoidance and path optimization capabilities in complex environments. On the other hand, PSO and GWO are less efficient in path planning when dealing with complex environments and are more likely to be interfered with by obstacles. In addition, from the convergence curve, the MIHHO algorithm can quickly converge and find a near-optimal path within a short number of iterations with less fluctuation, showing good stability and robustness. In contrast, other algorithms such as PSO and GWO converge more slowly and are easily affected by the trap of local optimization. Overall, MIHHO is more suitable for path planning tasks in complex environments.

Each algorithm was run independently on both maps 10 times, and the algorithm performance comparison is shown in Table 11. The MIHHO algorithm finds shorter optimal paths with an average path length of 29.70135 in a small-scale 20 × 20 environment. In comparison, the path length is reduced by 3.58% compared to GWO, 3.2% compared to SSA, 14.22% compared to WOA, 17.36% compared to PSO, 13.65% compared to GBO, 13.36% compared to SCSO, 5.52% compared to DBO, and 4.52% compared to HHO. Although SCSO has the lowest average number of iterations, its planned path is relatively long.

When the size of the raster map is 40 × 40, the average path length of MIHHO is 65.5203, which is significantly better than the other algorithms, indicating that MIHHO still maintains excellent path optimization in large-scale complex environments. The path length of MIHHO is reduced by 4.13% compared with GWO, 9.97% compared with SSA, 11.75% compared with WOA, 20.5% compared with PSO, 10.64% compared with GBO, 11.82% compared with SCSO, 8.38% compared with DBO, and 9.19% compared with HHO. Although MIHHO’s average number of iterations is 36.2, which is slightly higher than some of the algorithms, its significantly shorter paths indicate that it can achieve better results in relatively fewer iterations, fully demonstrating superior performance in complex problems. Despite the increased size and complexity of the map environment, the path planning ability of the MIHHO algorithm is not affected by the computational complexity and is still able to find shorter paths.

In maps with different specifications and complexities, the MIHHO algorithm presents certain advantages over the original HHO algorithm in the optimal path length, average path length, standard deviation of the path length, and average number of iteration metrics. It has better environmental applicability in different complex environments, and this advantage becomes more prominent as the size of the environmental map increases.

From these results, it can be seen that MIHHO is significantly better than the Harris Hawk Optimization algorithm as well as other common path planning algorithms in terms of the solution accuracy, convergence speed, and avoidance of falling into local optima through the introduction of multi-strategy improvement. Especially in complex environments, MIHHO shows stronger adaptability and efficiency, which verifies its practical application value and potential in path planning.

## 7. Conclusions

Aiming to solve the problems of poor optimization and low search stability in mobile robot path planning, this paper proposes a Multi-strategy Improved Harris Hawk Optimization (MIHHO) algorithm. Firstly, the double adaptive weight strategy is used to improve the exploration ability and exploitation ability of HHO to improve the convergence accuracy and speed of algorithms in path planning. Secondly, the Dimension Learning-Based Hunting (DLH) search strategy is used to enhance the ability of the Harris Hawk Algorithm to balance exploration and exploitation and maintain diversity. Then, position update strategy based on DBO algorithm is proposed to avoid falling into local optimal solutions during path planning. Through simulation experiments with 12 classical functions and CEC2022 test functions, the proposed MIHHO is verified to have strong global search capability and local search capability. Finally, path planning experiments are conducted in map environments of different sizes and complexities. The experimental results show that the proposed algorithm performs more prominently in terms of the optimality of the path length and stability than the other eight well-known algorithms. MIHHO demonstrates a faster convergence speed and higher stability, possesses good applicability and optimality-finding ability, and shows its potential in practical applications. This validates the effectiveness of the algorithm in global path planning problems for mobile robots.

This study proposes a Multi-strategy Improved Harris Hawk Optimization, mainly for the global path planning of robots, modeled by a two-dimensional raster. The experimental results show that the algorithm performs well in this area and has high practical value. However, there are still two major shortcomings: (1) This study focuses on the simulation of 2D motion scenes and does not consider the motion of UAVs in 3D space, so future research should be conducted in 3D environments to expand the applicability of the algorithm. (2) The experiments in this paper are mainly based on simulation, and future research needs to be carried out in real environments to enhance the practical application of the algorithm.

## Figures and Tables

**Table 1:** Classical benchmark functions.

Type	Function	Dim	Global Optima
Unimodal function	$F1(x)=\sum_{i=1}^{d}{x_{i}}^{2}$	30	0
	$F2(x)=\sum_{i=1}^{d}{\left(\sum_{j=1}^{d}x_{j}\right)}^{2}$	30	0
	$F3(x)=max_{i}\left\{\left|x_{i}\right|,1\leq i\leq d\right\}$	30	0
	$F4(x)=\sum_{i=1}^{d}i\cdot {x_{i}}^{4}+rand[0,1)$	30	0
Multimodal function	$F5(x)=\sum_{i=1}^{d}-x_{i}\sin(\sqrt{\left|x_{i}\right|})$	30	−418.9829 × Dim
	$F6(x)=\sum_{i=1}^{d}\left[{x_{i}}^{2}-10\cos(2x_{i}\pi )+10\right]$	30	0
	$F7(x)=-20\exp(-0.2\sqrt{\frac{1}{d}\sum_{i=1}^{d}{x_{i}}^{2}})-\exp\left[\frac{1}{d}\sum_{i=1}^{d}\cos(2x_{i}\pi )\right]+20+e$	30	0
	$F8(x)=\frac{1}{4000}\sum_{i=1}^{d}{x_{i}}^{2}-\prod_{i=1}^{d}\cos(\frac{x_{i}}{\sqrt{i}})+1$	30	0
Fixed-dimension multimodal function	$F9(x)={\left[0.002+\sum_{j=1}^{25}\frac{1}{j+\sum_{i=1}^{2}{\left(x_{i}-x_{ij}\right)}^{6}}\right]}^{-1}$	2	1
	$F10(x)=-{\sum_{i=1}^{5}\left[\left(x-a_{i}\right){\left(x-a_{i}\right)}^{T}+c_{i}\right]}^{-1}$	4	−10.1532
	$F11(x)=-{\sum_{i=1}^{7}\left[\left(x-a_{i}\right){\left(x-a_{i}\right)}^{T}+c_{i}\right]}^{-1}$	4	−10.4028
	$F12(x)=-{\sum_{i=1}^{10}\left[\left(x-a_{i}\right){\left(x-a_{i}\right)}^{T}+c_{i}\right]}^{-1}$	4	−10.5363

**Table 2:** Comparison of optimization results of different intelligent algorithms.

Functions	Index	GWO	SSA	WOA	PSO	GBO	SCSO	DBO	HHO	MIHHO
F1	Best	3.9079 × 10^−30^	0	7.9517 × 10^−87^	0.00063606	3.1733 × 10^−128^	3.0221 × 10^−125^	1.6001 × 10^−167^	3.0379 × 10^−113^	0
	Mean	8.8865 × 10^−28^	2.641 × 10^−54^	1.6 × 10^−73^	0.017306	1.8033 × 10^−116^	2.257 × 10^−112^	1.4317 × 10^−101^	1.2927 × 10^−91^	0
	Std	1.0723 × 10^−27^	1.4466 × 10^−53^	7.1291 × 10^−73^	0.034734	8.7403 × 10^−116^	1.1837 × 10^−111^	7.8418 × 10^−101^	7.0796 × 10^−91^	0
F2	Best	1.2306 × 10^−8^	1.4022 × 10^−92^	21,136.148	836.2801	1.9012 × 10^−108^	6.0921 × 10^−112^	1.8658 × 10^−160^	1.3875 × 10^−98^	0
	Mean	2.8545 × 10^−5^	5.1396 × 10^−27^	43,441.7047	2252.6295	7.9774 × 10^−93^	2.5836 × 10^−97^	3.9476 × 10^−78^	2.3464 × 10^−71^	0
	Std	9.7623 × 10^−5^	2.6469 × 10^−26^	14,283.7721	1888.0826	3.8353 × 10^−92^	1.4119 × 10^−96^	2.1622 × 10^−77^	1.2415 × 10^−70^	0
F3	Best	5.0592 × 10^−8^	1.2812 × 10^−119^	0.00020182	4.4928	8.7515 × 10^−61^	6.9719 × 10^−57^	4.3432 × 10^−78^	1.0308 × 10^−55^	0
	Mean	8.3153 × 10^−7^	4.3847 × 10^−29^	54.7644	6.8881	9.5192 × 10^−54^	2.4293 × 10^−50^	8.0166 × 10^−43^	4.9189 × 10^−48^	0
	Std	5.3132 × 10^−7^	2.0033 × 10^−28^	27.7913	1.652	3.7547 × 10^−53^	1.1926 × 10^−49^	4.3909 × 10^−42^	2.4996 × 10^−47^	0
F4	Best	0.00076823	0.00012883	0.00015767	0.023535	1.5371 × 10^−5^	2.8588 × 10^−6^	0.00017808	9.2355 × 10^−7^	7.6663 × 10^−7^
	Mean	0.0023435	0.0015127	0.0022641	0.050177	0.00075366	0.00016184	0.0010493	0.00017823	4.1231 × 10^−5^
	Std	0.001236	0.001253	0.0023919	0.020627	0.00048108	0.00030857	0.00078951	0.00018419	6.5041 × 10^−5^
F5	Best	−7188.2016	−9445.9825	−12,567.2869	−10,062.4719	−11,331.6109	−8739.4336	−12,569.4866	−12,569.4866	−12,569.4866
	Mean	−6047.4534	−8616.5616	−9923.8706	−8457.1427	−9141.0372	−6779.157	−12,561.848	−12,553.3976	−12,569.4241
	Std	753.6649	510.1863	1719.7314	717.7575	1007.4905	782.0046	39.5743	57.1789	0.11163
F6	Best	5.6843 × 10^−14^	0	0	33.8536	0	0	0	0	0
	Mean	2.464	0	5.1567	60.6847	0	0	0	0	0
	Std	3.9927	0	28.2444	20.048	0	0	0	0	0
F7	Best	6.839 × 10^−14^	8.8818 × 10^−16^	8.8818 × 10^−16^	0.012108	8.8818 × 10^−16^	8.8818 × 10^−16^	8.8818 × 10^−16^	8.8818 × 10^−16^	8.8818 × 10^−16^
	Mean	9.7759 × 10^−14^	8.8818 × 10^−16^	4.3225 × 10^−15^	0.78959	8.8818 × 10^−16^	8.8818 × 10^−16^	8.8818 × 10^−16^	8.8818 × 10^−16^	8.8818 × 10^−16^
	Std	1.7303 × 10^−14^	0	2.5523 × 10^−15^	0.7778	0	0	0	0	0
F8	Best	0	0	0	0.0019214	0	0	0	0	0
	Mean	0.0054766	0	3.700 × 10^−18^	0.033218	0	0	0.00057474	0	0
	Std	0.0097153	0	2.027 × 10^−17^	0.028653	0	0	0.003148	0	0
F9	Best	0.998	0.998	0.998	0.998	0.998	0.998	0.998	0.998	0.998
	Mean	5.0138	4.4439	2.9319	0.998	1.0311	3.5514	1.4558	1.6249	0.998
	Std	4.2031	4.9767	2.9653	7.1417 × 10^−17^	0.18148	3.4054	1.8284	1.2557	3.4985 × 10^−11^
F10	Best	−10.1531	−10.1532	−10.1526	−10.1532	−10.1532	−10.1532	−10.1532	−10.0896	−10.1529
	Mean	−9.3149	−7.9441	−8.6934	−6.5652	−8.4539	−5.7364	−7.7898	−5.1382	−10.1479
	Std	2.2163	2.5694	2.4565	3.5141	2.4443	1.762	2.5662	1.0359	0.0046385
F11	Best	−10.4024	−10.4029	−10.4012	−10.4029	−10.4029	−10.4029	−10.4029	−10.2685	−10.4026
	Mean	−10.4011	−8.6312	−7.1244	−8.5207	−8.4086	−6.1973	−8.2877	−5.594	−10.3967
	Std	0.0011786	2.5485	2.9416	3.2195	2.6768	2.6959	2.6305	1.5557	0.0061323
F12	Best	−10.5362	−10.5364	−10.5349	−10.5364	−10.5364	−10.5364	−10.5364	−5.1285	−10.5363
	Mean	−10.2639	−8.7338	−6.2514	−8.2255	−7.7551	−6.9006	−8.0298	−5.122	−10.5312
	Std	1.4812	2.5929	3.1803	3.4083	2.8594	2.8879	2.7245	0.0096613	0.0054868
Estimation	Mean	5.08	4.33	5.42	5.83	3	4.17	3.75	4.5	1.08
	Rank	7	5	8	9	2	4	3	6	1

**Table 3:** Results of Wilcoxon rank sum test for classical functions.

Functions	GWO	SSA	WOA	PSO	GBO	SCSO	DBO	HHO
F1	1.2118 × 10^−12^	4.5736 × 10^−12^	1.2118 × 10^−12^	1.2118 × 10^−12^	1.2118 × 10^−12^	1.2118 × 10^−12^	1.2118 × 10^−12^	1.2118 × 10^−12^
F2	1.2118 × 10^−12^	1.2118 × 10^−12^	1.2118 × 10^−12^	1.2118 × 10^−12^	1.2118 × 10^−12^	1.2118 × 10^−12^	1.2118 × 10^−12^	1.2118 × 10^−12^
F3	1.2118 × 10^−12^	4.5736 × 10^−12^	1.2118 × 10^−12^	1.2118 × 10^−12^	1.2118 × 10^−12^	1.2118 × 10^−12^	1.2118 × 10^−12^	1.2118 × 10^−12^
F4	3.0199 × 10^−11^	8.891 × 10^−10^	3.4742 × 10^−10^	3.0199 × 10^−11^	4.1997 × 10^−10^	0.00055611	8.1014 × 10^−10^	0.00076973
F5	3.0199 × 10^−11^	3.0199 × 10^−11^	3.0199 × 10^−11^	3.0199 × 10^−11^	3.0199 × 10^−11^	3.0199 × 10^−11^	3.0199 × 10^−11^	1.8682 × 10^−5^
F6	4.5164 × 10^−12^	NaN	NaN	1.2118 × 10^−12^	NaN	NaN	0.33371	NaN
F7	1.1359 × 10^−12^	NaN	1.1585 × 10^−8^	1.2118 × 10^−12^	NaN	NaN	NaN	NaN
F6	0.0055843	NaN	0.1608	1.2118 × 10^−12^	NaN	NaN	NaN	NaN
F8	4.9752 × 10^−11^	0.075607	1.7769 × 10^−10^	6.3188 × 10^−12^	5.1812 × 10^−12^	2.1959 × 10^−7^	8.6748 × 10^−6^	2.0338 × 10^−9^
F9	7.6588 × 10^−5^	0.0072169	1.3853 × 10^−6^	0.0078403	0.0075506	8.4848 × 10^−9^	1.7273 × 10^−5^	3.0199 × 10^−11^
F10	4.9426 × 10^−5^	0.025536	3.5201 × 10^−7^	0.0015453	0.000267	9.5139 × 10^−6^	7.6152 × 10^−5^	3.0199 × 10^−11^
F11	0.00022539	5.3435 × 10^−5^	1.85 × 10^−8^	0.0074461	0.0075566	6.765 × 10^−5^	0.0039086	3.0199 × 10^−11^
F12	1.2118 × 10^−12^	4.5736 × 10^−12^	1.2118 × 10^−12^	1.2118 × 10^−12^	1.2118 × 10^−12^	1.2118 × 10^−12^	1.2118 × 10^−12^	1.2118 × 10^−12^
+/=/−	12/0/0	8/3/1	10/1/1	12/0/0	9/3/0	9/3/0	9/2/1	9/3/0

**Table 4:** CEC2022 test function details.

Type	Function	Descriptive	Global Optima
UF	F13(CEC1)	Shifted and Full Rotated Zakharov Function	300
BF	F14(CEC2)	Shifted and Full Rotated Rosenbrock’s Function	400
	F15(CEC3)	Shifted and Full Rotated Expanded Schaffer’s f6	600
	F16(CEC4)	Shifted and Full Rotated Non-Continuous Rastrigin’s Function	800
	F17(CEC5)	Shifted and Full Rotated Levy Function	900
HF	F18(CEC6)	Hybrid Function 1 (N = 3)	1800
	F19(CEC7)	Hybrid Function 2 (N = 6)	2000
	F20(CEC8)	Hybrid Function 3 (N = 5)	2200
CF	F21(CEC9)	Composition Function 1 (N = 5)	2300
	F22(CEC10)	Composition Function 2 (N = 4)	2400
	F23(CEC11)	Composition Function 3 (N = 5)	2600
	F24(CEC12)	Composition Function 4 (N = 6)	2700

**Table 5:** Comparison of CEC2022 test function optimization results.

Function	Index	GWO	SSA	WOA	PSO	GBO	SCSO	DBO	HHO	MIHHO
F13	Best	339.7353	300	12,561.1581	300	300	418.1413	300.8018	358.61	300
	Mean	2576.2897	303.0602	30,798.4928	300.0367	300.0183	3289.2294	2138.4791	1086.4727	300.0177
	Std	2333.0803	10.8327	14,457.3781	0.15351	0.22764	2151.6108	1964.0605	493.6898	0.0039427
F14	Best	404.1306	400.0093	404.4626	400.3851	400.1968	402.1813	404.9318	400.2839	400.0019
	Mean	435.4307	412.865	467.277	436.4721	408.6839	431.3265	439.2044	463.4548	408.0651
	Std	26.413	23.3158	88.2943	66.9228	24.9756	28.6752	56.23	62.5455	12.6514
F15	Best	600.0776	600.0004	620.7523	600	600.0028	603.4417	601.9854	622.115	600.1547
	Mean	601.8192	604.5389	639.8656	600.4853	600.86	617.4314	613.2101	640.0181	602.1263
	Std	4.5103	8.6267	13.0016	1.0975	1.289	11.6609	8.589	11.4329	0.97759
F16	Best	806.6137	806.9647	812.4975	803.9798	808.9546	807.6149	807.9637	815.1933	809.9519
	Mean	815.9944	828.9864	839.6426	815.8086	822.6187	828.1278	832.8943	826.6859	833.3066
	Std	7.7395	9.968	17.0224	8.1472	10.5987	8.5899	13.6251	6.5979	8.9145
F17	Best	900.25	936.3489	1075.8395	900	900	906.5558	906.9326	1204.7792	943.4538
	Mean	943.3298	1388.5225	1596.4899	900.4999	915.3015	1148.2269	1023.872	1462.7634	1319.221
	Std	86.2365	177.0459	480.2666	0.7359	26.8651	183.5889	140.3544	141.8046	184.9728
F18	Best	2676.966	1909.9082	2207.2638	1890.7644	1831.8326	2157.598	1996.5885	2365.7332	1874.7405
	Mean	6728.5109	4198.761	5769.3454	5313.3755	3430.7368	5323.3402	5593.4363	7545.2141	3183.9722
	Std	2217.4785	1545.6812	2891.1287	2227.0454	1923.2512	1990.6186	2590.603	6485.6816	1758.3396
F19	Best	2021.9415	2005.5998	2046.625	2000.6243	2005.5649	2022.2196	2022.0505	2040.2114	2001.328
	Mean	2031.741	2033.3058	2082.5441	2019.0671	2023.298	2043.1307	2045.0321	2090.901	2020.5375
	Std	7.1208	26.1242	27.3671	8.0415	8.4338	17.1861	30.7455	34.6306	6.2211
F20	Best	2207.6728	2220.1155	2226.4034	2200.8093	2206.2251	2221.6339	2221.6866	2223.7919	2204.9731
	Mean	2241.2538	2233.7783	2237.3402	2254.3118	2224.2991	2227.9122	2232.9896	2237.8373	2220.9942
	Std	40.2969	36.4304	11.0016	55.3187	4.3037	3.8658	20.9083	9.0343	3.1009
F21	Best	2529.3391	2529.2844	2551.0113	2529.2844	2529.2844	2529.2989	2529.2844	2543.0909	2529.2863
	Mean	2579.7157	2548.8938	2624.5304	2535.7198	2529.2844	2573.3561	2563.494	2621.089	2534.1854
	Std	37.2357	50.7938	50.8682	27.016	4.3879 × 10^−13^	30.7537	52.0602	38.9846	26.8255
F22	Best	2500.3161	2500.2153	2500.7107	2500.3665	2500.3319	2500.3769	2500.5935	2500.5952	2500.3736
	Mean	2597.1597	2642.1248	2770.7768	2583.4666	2558.2117	2565.6277	2555.9037	2697.673	2533.1447
	Std	136.8643	209.9238	424.5043	79.5016	62.8245	66.5665	68.7745	303.6366	74.2088
F23	Best	2603.886	2600	2737.3267	2600	2600	2606.164	2600	2611.1604	2600
	Mean	3005.905	2816.3805	3039.2047	2783.5197	2826.79	2899.8734	2771.6167	2866.442	2727.1559
	Std	168.7909	135.5907	338.7632	131.4767	129.7348	178.8386	111.1563	155.1139	127.0772
F24	Best	2863.5019	2862.5674	2870.2711	2862.4421	2861.4049	2863.7479	2863.6612	2868.8918	2859.6596
	Mean	2873.0944	2880.9625	2915.3618	2874.3854	2871.5042	2873.5491	2882.3811	2944.4871	2872.7128
	Std	13.1856	31.5485	46.1435	16.813	20.0176	11.2892	24.5016	56.6887	12.0736
Estimation	Mean	4.92	4.92	8.33	3.5	2.33	5.33	5.25	7.5	2.5
	Rank	4	4	9	3	1	7	6	8	2

**Table 6:** Results of Wilcoxon rank sum test for CEC2022 test functions.

Function	GWO	SSA	WOA	PSO	GBO	SCSO	DBO	HHO
F1	1.2118 × 10^−12^	4.5736 × 10^−12^	1.2118 × 10^−12^	1.2118 × 10^−12^	1.2118 × 10^−12^	1.2118 × 10^−12^	1.2118 × 10^−12^	1.2118 × 10^−12^
F2	1.2118 × 10^−12^	1.2118 × 10^−12^	1.2118 × 10^−12^	1.2118 × 10^−12^	1.2118 × 10^−12^	1.2118 × 10^−12^	1.2118 × 10^−12^	1.2118 × 10^−12^
F3	1.2118 × 10^−12^	4.5736 × 10^−12^	1.2118 × 10^−12^	1.2118 × 10^−12^	1.2118 × 10^−12^	1.2118 × 10^−12^	1.2118 × 10^−12^	1.2118 × 10^−12^
F4	3.0199 × 10^−11^	8.891 × 10^−10^	3.4742 × 10^−10^	3.0199 × 10^−11^	4.1997 × 10^−10^	0.00055611	8.1014 × 10^−10^	0.00076973
F5	3.0199 × 10^−11^	3.0199 × 10^−11^	3.0199 × 10^−11^	3.0199 × 10^−11^	3.0199 × 10^−11^	3.0199 × 10^−11^	3.0199 × 10^−11^	1.8682 × 10^−5^
F6	4.5164 × 10^−12^	NaN	NaN	1.2118 × 10^−12^	NaN	NaN	0.33371	NaN
F7	1.1359 × 10^−12^	NaN	1.1585 × 10^−8^	1.2118 × 10^−12^	NaN	NaN	NaN	NaN
F6	0.0055843	NaN	0.1608	1.2118 × 10^−12^	NaN	NaN	NaN	NaN
F8	4.9752 × 10^−11^	0.075607	1.7769 × 10^−10^	6.3188 × 10^−12^	5.1812 × 10^−12^	2.1959 × 10^−7^	8.6748 × 10^−6^	2.0338 × 10^−9^
F9	7.6588 × 10^−5^	0.0072169	1.385 × 10^−6^	0.0078403	0.0075506	8.4848 × 10^−9^	1.7273 × 10^−5^	3.0199 × 10^−11^
F10	4.9426 × 10^−5^	0.025536	3.5201 × 10^−7^	0.0015453	0.000267	9.5139 × 10^−6^	7.6152 × 10^−5^	3.0199 × 10^−11^
F11	0.00022539	5.3435 × 10^−5^	1.85 × 10^−8^	0.0074461	0.0075566	6.765 × 10^−5^	0.0039086	3.0199 × 10^−11^
F12	1.2118 × 10^−12^	4.5736 × 10^−12^	1.2118 × 10^−12^	1.2118 × 10^−12^	1.2118 × 10^−12^	1.2118 × 10^−12^	1.2118 × 10^−12^	1.2118 × 10^−12^
+/=/−	12/0/0	8/3/1	10/1/1	12/0/0	9/3/0	9/3/0	9/2/1	9/3/0

**Table 7:** Comparison of different improved HHO search results.

Function	Index	NCHHO	LHHO	LMHHO	MIHHO
F1	Best	4.3835 × 10^−239^	8.3093 × 10^−166^	0	0
	Mean	2.1917 × 10^−240^	2.8468 × 10^−145^	0	0
	Std	0	9.8053 × 10^−145^	0	0
F2	Best	2.1941 × 10^−249^	6.6287 × 10^−135^	0	0
	Mean	1.8669 × 10^−214^	1.2616 × 10^−100^	0	0
	Std	0	6.8972 × 10^−100^	0	0
F3	Best	8.0046 × 10^−131^	1.6516 × 10^−82^	1.849 × 10^−249^	0
	Mean	5.6251 × 10^−119^	8.8838 × 10^−71^	2.316 × 10^−236^	0
	Std	2.3441 × 10^−118^	4.7576 × 10^−70^	0	0
F4	Best	1.0575 × 10^−7^	9.3114 × 10^−6^	4.037 × 10^−7^	1.0246 × 10^−6^
	Mean	0.0001236	9.2186 × 10^−5^	9.3295 × 10^−5^	1.8966 × 10^−5^
	Std	9.911 × 10^−5^	5.3666 × 10^−5^	9.2981 × 10^−5^	1.6225 × 10^−5^
F5	Best	−12,569.4817	−12,569.4865	−12,569.487	−12,569.4866
	Mean	−12,501.4776	−12,569.4717	−12,569.214	−12,569.4124
	Std	177.6465	0.21166	1.0492	0.12898
F6	Best	0	0	0	0
	Mean	0	0	0	0
	Std	0	0	0	0
F7	Best	8.8818 × 10^−16^	8.8818 × 10^−16^	8.8818 × 10^−16^	8.8818 × 10^−16^
	Mean	8.8818 × 10^−16^	8.8818 × 10^−16^	8.8818 × 10^−16^	8.8818 × 10^−16^
	Std	0	0	0	0
F8	Best	0	0	0	0
	Mean	0	0	0	0
	Std	0	0	0	0
F9	Best	0.998	0.998	0.998	0.998
	Mean	1.7916	1.0311	0.998	0.998
	Std	1.4977	0.18148	1.44 × 10^−10^	1.33 × 10^−11^
F10	Best	−10.1025	−10.1532	−5.0548	−10.1532
	Mean	−8.2697	−9.1315	−4.9995	−10.1473
	Std	2.0236	2.073	0.11759	0.0082563
F11	Best	−10.3122	−10.4026	−5.0876	−10.4027
	Mean	−7.8291	−9.3316	−5.0278	−10.3978
	Std	2.453	2.1583	0.10179	0.0043626
F12	Best	−10.4924	−10.5364	−5.1278	−10.5363
	Mean	−7.2542	−9.6282	−5.0867	−10.5309
	Std	2.496	2.0468	0.086013	0.0053248

**Table 8:** Different strategies for HHO.

	Double Adaptive Weight Strategy	DLH Search Strategy	Position Update Strategy Based on DBO Algorithm
HHO	0	0	0
HHO1	1	0	0
HHO2	0	1	0
HHO3	0	0	1
HHO12	1	1	0
HHO13	1	0	1
HHO23	0	1	1

**Table 9:** Comparison of optimization results of ablation experiments.

Function	Index	HHO	HHO1	HHO2	HHO3	HHO12	HHO13	HHO23
F1	Best	3.3253 × 10^−112^	8.9003 × 10^−171^	0	8.0823 × 10^−227^	0	2.7309 × 10^−271^	0
	Mean	1.5801 × 10^−96^	5.7737 × 10^−135^	4.9407 × 10^−324^	0	0	2.112 × 10^−253^	0
	Std	8.343 × 10^−96^	1.0541 × 10^−135^	0	3.8473 × 10^−207^	0	0	0
F2	Best	1.2599 × 10^−96^	2.0311 × 10^−148^	2.0311 × 10^−148^	4.0299 × 10^−239^	0	4.1191 × 10^−239^	0
	Mean	6.6397 × 10^−66^	1.1736 × 10^−122^	2.1428 × 10^−123^	0	0	1.5794 × 10^−203^	1.9179 × 10^−282^
	Std	3.6367 × 10^−65^	2.1428 × 10^−123^	1.1736 × 10^−122^	6.169 × 10^−169^	0	0	0
F3	Best	2.2842 × 10^−57^	3.2572 × 10^−85^	2.9095 × 10^−165^	1.1407 × 10^−105^	4.5555 × 10^−222^	1.2775 × 10^−135^	2.9634 × 10^−176^
	Mean	7.8555 × 10^−50^	1.015 × 10^−72^	9.6934 × 10^−146^	9.1514 × 10^−98^	1.5838 × 10^−202^	7.7083 × 10^−123^	3.2638 × 10^−156^
	Std	2.9179 × 10^−49^	1.89 × 10^−73^	5.2825 × 10^−145^	1.6848 × 10^−98^	0	4.2008 × 10^−122^	1.7562 × 10^−155^
F6	Best	0	0	0	0	0	0	0
	Mean	0	0	0	0	0	0	0
	Std	0	0	0	0	0	0	0
F8	Best	0	0	0	0	0	0	0
	Mean	0	0	0	0	0	0	0
	Std	0	0	0	0	0	0	0
F13	Best	499.5464	451.3108	300.0151	405.3196	300.0091	300.073	300.0109
	Mean	1054.6712	723.4408	304.8567	876.3008	302.4262	301.6767	300.0219
	Std	450.29	296.5269	13.9405	323.1338	70.5143	2.0059	0.007624
F14	Best	426.1614	412.2925	404.3665	405.4668	400.2645	400.092	400.0791
	Mean	482.2627	465.128	473.499	441.9414	451.3182	414.1834	424.0303
	Std	46.4494	67.3456	74.5752	56.6134	55.7907	20.6095	34.091
F18	Best	2397.4281	2011.1804	2056.762	2123.1912	1972.6731	1990.5764	1906.2921
	Mean	7016.2047	4514.9281	3871.4013	3963.7292	3275.5863	3392.9889	3280.4198
	Std	6090.2478	4452.2142	2301.3651	3065.6397	3052.8942	2258.0825	2056.7581
F21	Best	2585.9431	2529.2851	2540.4439	2552.0027	2529.3682	2529.2844	2533.6741
	Mean	2696.4308	2593.1123	2668.2599	2635.4749	2598.81	2589.1342	2619.374
	Std	40.9474	49.2524	31.7577	43.6345	51.2598	37.2641	46.626

**Table 10:** Comparative experimental results of path planning in simple environments.

Map Size	Algorithm	Path Length			Iterations		
		Best	Mean	Std	Best	Mean	Std
20 × 20	GWO	28.1302	28.99975	0.730980313	23	47.2	22.55758064
	SSA	28.1749	29.31758	1.320170692	2	25.5	26.56752403
	WOA	29.0814	31.83472	1.953097743	1	13	10
	PSO	28.1302	32.25132	2.554425596	41	67.3	18.49954954
	GBO	28.1302	29.73836	1.201097738	48	69	16.91153453
	SCSO	30.3603	33.01106	1.847597231	1	14	19.9276469
	DBO	28.1302	28.92537	0.677979036	19	48.9	22.323132
	HHO	28.1302	28.92959	0.885522541	10	45.6	31.52142129
	MIHHO	27.8279	28.35375	0.340219678	9	17.6	9.570788891
40 × 40	GWO	60.259	64.4877	4.299023495	26	68.6	22.52998003
	SSA	64.9702	71.00163	2.457679449	7	40.7	23.73955911
	WOA	70.4079	75.30136	2.098619263	5	16.7	11.83262909
	PSO	73.1966	112.78083	20.932749	86	94.4	5.738757124
	GBO	72.5571	74.09069	1.725264679	6	41.6	22.30196802
	SCSO	73.5807	75.97282	1.057445971	2	7.4	3.306559138
	DBO	61.3757	67.25799	3.752098025	15	58.3	23.39539366
	HHO	69.2777	71.36853	1.445605752	7	22.1	16.78921612
	MIHHO	58.2233	61.05562	1.556186577	13	32.2	18.20134305

**Table 11:** Comparative experimental results of path planning in complex environments.

Map Size	Algorithm	Path Length			Iterations		
		Best	Mean	Std	Best	Mean	Std
20 × 20	GWO	28.9969	30.80291	1.187916685	10	18.1	5.78215646
	SSA	28.8569	30.68388	1.534795537	21	42.5	22.62864458
	WOA	32.2587	34.62525	1.307826451	1	11.3	7.616502551
	PSO	30.9325	35.941	2.579778856	1	11.9	18.02128371
	GBO	32.0805	34.39496	1.656553955	1	47.1	29.69268522
	SCSO	31.964	34.2809	1.702263173	1	10.5	15.27707069
	DBO	30.0285	31.43774	1.423925406	6	21.1	16.07240561
	HHO	30.1685	31.10808	1.208440051	14	40.2	30.17283547
	MIHHO	28.8569	29.70135	0.793536696	4	15.5	8.2360994
40 × 40	GWO	67.0996	70.99656	3.51914798	14	53.7	29.63125227
	SSA	75.0711	75.59832	0.185246225	7	28.8	17.32563932
	WOA	76.2426	77.1213	0.497834272	3	6	3.771236166
	PSO	80.6919	85.61078	3.460530845	1	19.4	24.42312201
	GBO	74.8929	76.16624	0.784418269	12	45.2	22.14497686
	SCSO	75.6569	77.18406	0.563175905	1	3.4	2.319003617
	DBO	67.2777	74.28681	2.734354672	3	41.1	29.58396713
	HHO	70.5138	74.94902	1.612339986	6	26.5	28.43413442
	MIHHO	65.5203	68.06217	1.030074189	28	36.2	6.908931418

## Data Availability

The original contributions presented in the study are included in the article material, further inquiries can be directed to the corresponding author.
